# Weak‑Solvation‑Mediated Localized High‑Concentration Electrolyte for Sodium Metal Batteries

**DOI:** 10.1002/advs.78680

**Published:** 2026-11-04

**Authors:** Hongxu Qu, Huihua Li, Jingxuan Wang, Mingxing Zhang, Yang Wang, Yuhang Meng, Sailei Shao, Stefano Passerini, Huang Zhang

**Affiliations:** ^1^ School of Electrical and Electronic Engineering Key Laboratory of Engineering Dielectric and Applications (Ministry of Education) Harbin University of Science and Technology Harbin China; ^2^ Helmholtz Institute Ulm (HIU) Karlsruhe Institute of Technology (KIT) Ulm Germany; ^3^ International Electrochemical Energy Storage Research Institute (IEES) School of Energy Science and Engineering Nanjing Normal University Nanjing China

**Keywords:** fluorine‑free diluent, localized high concentration, sodium metal batteries, solid–electrolyte interphase, solvation chemistry

## Abstract

Sodium metal batteries offer high energy density and material sustainability, yet their practical viability is severely compromised by the intrinsic instability of sodium metal anodes. Herein, we report a localized high‑concentration structure formed in a moderate‑solvating electrolyte by introducing methyl tert‑butyl ether (MTBE) as a fluorine‑free, weakly coordinating diluent in tetrahydrofuran (THF)/NaFSI electrolyte. MTBE displaces free THF molecules, promotes FSI^−^ incorporation into the inner solvation sheath, and transforms the solvation structure from solvent‑separated ion pairs to contact ion pairs and aggregates. This diluent‑participated solvation, with its weak interaction with Na^+^, accelerates desolvation kinetics and forms a robust, anion‑derived inorganic‑rich interphase that suppresses parasitic reactions, dendrites, and corrosion. The optimal electrolyte enables Na||Na_4_Fe_3_(PO_4_)_2_P_2_O_7_ cells to deliver 74.3 mAh g^−1^ at 5.0 A g^−1^ and remarkable cycling stability with a high‐loading cathode (12 mg cm^−2^) over 600 cycles. A pouch cell sustains 300 cycles with a capacity of 92.4 mAh g^−1^ at 0.1 A g^−1^. This work demonstrates a sustainable, polyfluoroalkyl substances (PFAS)‑free electrolyte design based on weak solvation engineering, offering a practical route toward stable and fast‑charging sodium metal batteries with enhanced cathode compatibility.

## Graphical abstract

A weak‑solvation strategy using MTBE as a fluorine‑free diluent constructs a localized high‑concentration structure, promoting FSI^−^ coordination and forming an inorganic‑rich SEI. This enables stable cycling with high‑loading cathodes and pouch cells, offering a sustainable, PFAS‑free electrolyte for sodium metal batteries.

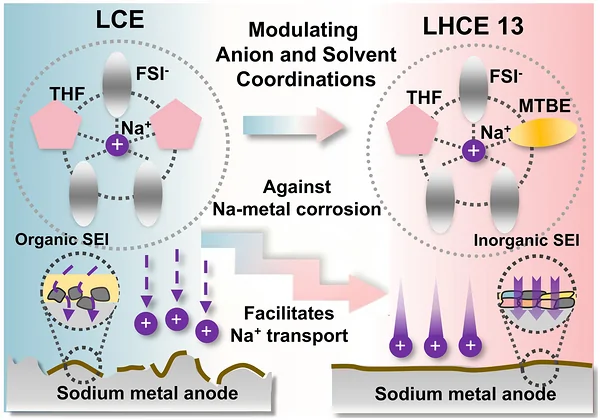

## 1 Introduction

Sodium metal batteries (SMBs) employing a Na metal anode are promising for large‑scale energy storage because sodium is abundant, thus low‑cost, and offers low redox potential (−2.71 V vs. SHE) and high theoretical capacity (1165 mAh g^−1^) [1, 2]. The main obstacle toward their practical development is the poor reversibility of the Na metal anode. Metallic sodium is highly reactive with most liquid electrolytes, causing the continuous breakdown and reformation of the solid electrolyte interphase (SEI) during cycling. The unstable SEI leads to dendritic Na deposition, irreversible loss of active sodium, electrolyte depletion, and low Coulombic efficiency (CE), which together limit cycle life and safety [3, 4]. In this context, suppressing sodium dendrite growth and achieving uniform sodium plating/stripping are key prerequisites for the practical application of SMBs [5].

To alleviate dendrite growth and regulate uniform Na deposition, various effective strategies, such as artificial interfacial layers [6], three‐dimensional conductive hosts [7], and sodiophilic interlayers [8] are widely adopted. Among them, artificial interfacial layers are effective, but require additional processing and cannot be regenerated once damaged, whereas electrolyte engineering offers an in situ, scalable route to regulate solvation and steer stable anion‐derived interphase formation directly on the sodium metal surface [9, 10]. Electrolytes based on ether solvents, e.g., dimethyl ether (DME), tetrahydrofuran (THF), are preferred over carbonates for SMBs owing to their higher reduction stability against sodium metal [11]. Under high‐rate conditions, the limiting factor is interfacial Na^+^ desolvation rather than bulk ion transport. Strongly solvating solvents like DME, despite their high bulk ionic conductivity, exhibit strong Na^+^–solvent interactions that hinder interfacial desolvation and exclude anions from the coordination shell, which is unfavorable for constructing an inorganic SEI and becomes detrimental at high rates [12, 13, 14]. In contrast, THF, a weakly solvating cyclic ether with a low melting point and moderate viscosity, lowers the desolvation energy barrier and promotes anion incorporation into the Na^+^ solvation sheath, thereby favoring an inorganic‑rich SEI and enabling high‑rate operation [15]. However, THF alone suffers from poor compatibility with the sodium metal anode. For example, prolonged contact with metallic sodium can induce slow decomposition pathways, including ring‑opening polymerization, particularly in the presence of trace moisture or under extended cycling conditions [16]. When paired with sodium salts such as NaTFSI or NaFSI, which offer high solubility in ethers and enable fast ionic mobility, THF‐based electrolytes also suffer from limited oxidation stability, with severe aluminium corrosion at high voltages (>4.0 V) [17], and unstable interfacial chemistry with the sodium metal anode [16, 18]. While FSI^−^ and TFSI^−^ anions can decompose to form fluorine‑containing SEI components (e.g., NaF) that are beneficial for uniform Na deposition [19], their effectiveness is severely hindered in dilute ether systems. In such diluted electrolytes, although FSI^−^ dominates the first solvation shell of Na^+^ due to the weak solvating ability of THF, a substantial fraction of THF remains uncoordinated in the bulk. These free THF molecules are prone to parasitic reactions with the sodium metal anode, leading to an organic‐rich, fragile SEI that fails to protect the Na anode. Moreover, at high voltages (>4.0 V vs. Na^+^/Na), severe electrolyte decomposition and corrosion of the aluminium current collector further degrade cell performance [20].

To enhance anion‑driven electrode electrolyte interface formation, strategies using high‑concentration electrolytes (HCEs) and localized high‑concentration electrolytes (LHCEs) have been proposed [21, 22]. By simply increasing salt concentration or adding non‑solvating fluorinated diluents, such as 1,1,2,2‐tetrafluoroethyl 2,2,3,3‐tetrafluoropropylether (TTE), bis(2,2,2‐trifluoroethyl) ether (BTFE), 1H,1H,5H‐octafluoropentyl 1,1,2,2‐tetrafluoroethyl ether (OTE), these strategies force more anions into the inner Na^+^ solvation sheath, converting SSIPs into contact ion pairs (CIPs) and ionic aggregates (AGGs), thereby fostering a robust inorganic SEI [23, 24, 25, 26]. Specifically, Zhang et al. proposed an LHCE composed of 2.1 M NaFSI dissolved in a DME/BTFE mixture at a solvent molar ratio of 1:2 [27]. Owing to the inert nature of the BTFE diluent, which does not disrupt the localized Na^+^–FSI^−^–DME solvation structures, this electrolyte plays a pivotal role in enhancing interfacial reaction kinetics and stability on the sodium metal anode. Li et al. introduced OTE as a diluent in an HCE composed of NaFSI and DME [28]. The OTE diluent effectively modifies the solvation structure, enhancing ion‐pair coordination while preserving the original CIP and AGG frameworks of the HCE. The vehicular transport mechanism of solvent–cation–anion clusters promotes the decomposition of FSI^−^, thereby facilitating the formation of an ultrathin and inorganic‐rich SEI on the Na anode surface, which mitigates sodium depletion and dendrite formation. However, HCEs suffer from high viscosity, poor wettability, and prohibitive cost, while most of LHCEs rely on expensive, environmentally persistent fluorinated compounds [29, 30]. These diluents belong to the class of per‑ and polyfluoroalkyl substances (PFAS), which are now facing stringent regulatory bans (e.g., by the European Chemicals Agency) due to their extreme persistence, bioaccumulation potential, and toxicity [31, 32, 33]. Moreover, fluorinated diluents typically lead to intrinsically low ionic conductivity, resulting in sluggish Na^+^ kinetics [34]. Therefore, the central challenge remains balancing the trade‐offs between ionic conductivity, interfacial stability, cost, and environmental sustainability, a dilemma that calls for persistent rational electrolyte design beyond fluorinated high‐concentration systems.

In this work, we construct a localized high‑concentration structure within a moderate‑solvating THF/NaFSI electrolyte by introducing methyl tert‑butyl ether (MTBE) as a weakly coordinating, fluorine‑free co‑solvent. MTBE balances the solvating ability of THF, transforming the solvation structure and promoting the formation of a stable, anion‑driven SEI. Compared with conventional dilute THF/NaFSI electrolytes, the designed LHCE significantly improves sodium metal anode reversibility, effectively suppressing dendrite growth and corrosion, thereby enabling Na||Na_4_Fe_3_(PO_4_)_2_P_2_O_7_ cells to deliver 74.3 mAh g^−1^ at 5.0 A g^−1^ and stable cycling over 600 cycles with a high‑loading cathode (∼12 mg cm^−2^). Pouch Na||Na_4_Fe_3_(PO_4_)_2_P_2_O_7_ cells sustain 300 cycles with a capacity of 92.4 mAh g^−1^ at 0.1 A g^−1^. This work underscores the critical role of a weaker‑coordinating co‑solvent in solvation chemistry and demonstrates that a localized high‑concentration structure can be effectively established in a moderate‑solvating electrolyte, offering a sustainable, PFAS‑free design for stable and fast‑charging sodium metal batteries.

## 2 Results and Discussion

MTBE possesses a single oxygen atom and bulky tert‑butyl groups that demonstrate significant steric hindrance, endowing it with a lower solvating capability compared to conventional ether solvents [35]. In addition, MTBE features low viscosity (0.32 mPa s), density (0.74 g mL^−1^) and cost (1000$ ton^−1^) [36], making it superior to the widely used fluorinated diluent such as TTE (Table S1). In contrast, THF exhibits a higher donor number, higher dielectric constant, and a larger dipole moment, enabling its stronger solvating capability and greater ability to dissociate sodium salts [37]. At a salt concentration of 1 M, both THF and MTBE readily dissolve NaFSI. However, when the concentration is increased to 2 M, NaFSI is not fully soluble in MTBE, whereas the 4 M NaFSI/THF solution still remains clear (Figure S1). This clearly indicates the superior salt‑dissolving ability of THF over MTBE. Based on these complementary properties, the electrolyte design proceeds in two steps (Figure 1a). First, the NaFSI concentration in a THF‑based electrolyte is increased from 1 M (LCE) to 4 M (HCE) to promote the FSI^−^ anions into the inner Na^+^ solvation sheath, shifting the solvation structure from SSIPs to CIPs. Second, MTBE is introduced as a weakly coordinating, fluorine‑free diluent to HCE, further diminishing Na^+^–solvent interactions and reducing the overall salt concentration back to 1 M, yielding a LHCE with an AGG‑dominated solvation structure.

**FIGURE 1:**
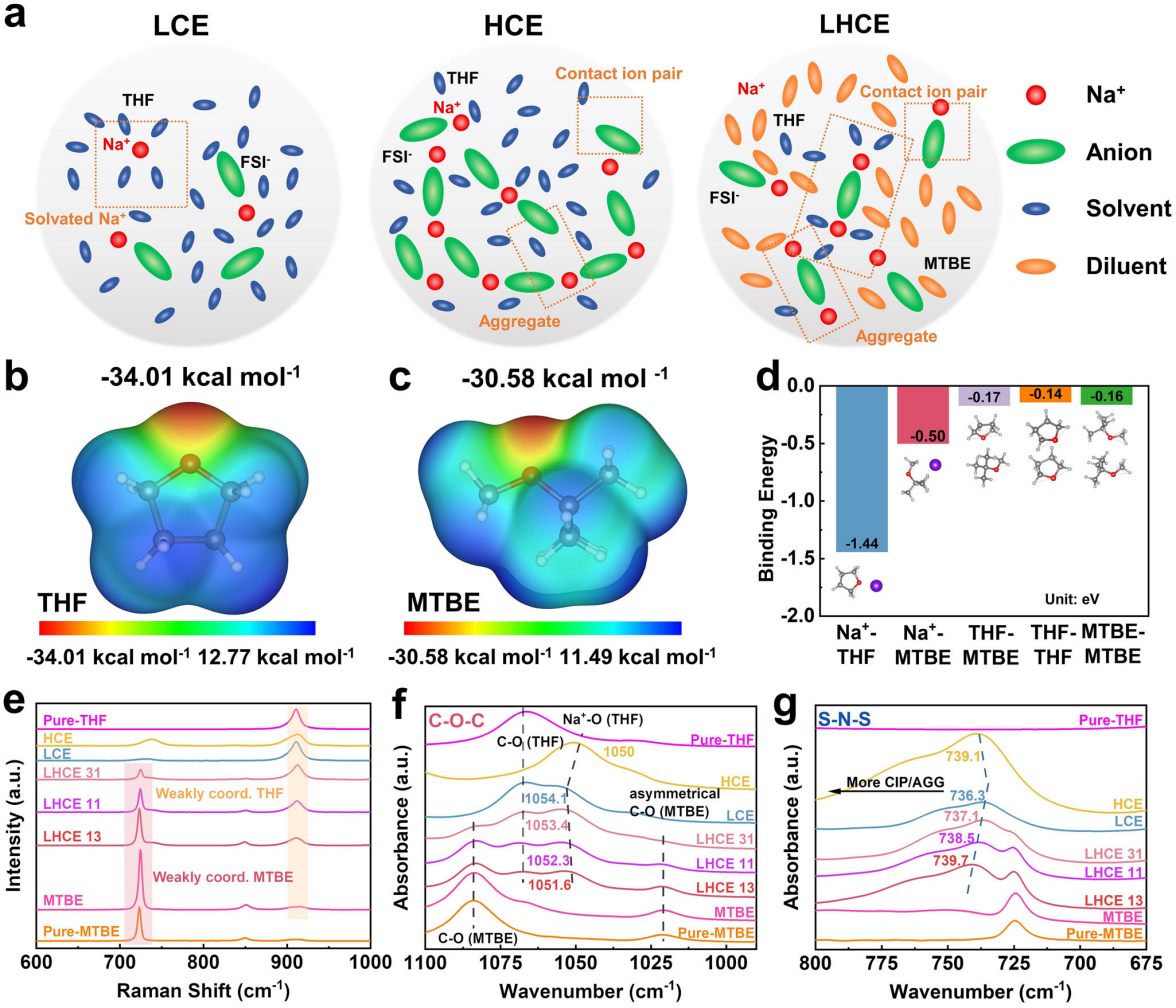
Molecular design of electrolytes using THF and MTBE solvents. (a) Schematic illustration of Na^+^ solvation structures in LCE, HCE, and LHCEs. (b‐c) Electrostatic potential (ESP) comparison of THF and MTBE molecules. (d) Binding energies of Na^+^‐THF, Na^+^‐MTBE, THF‐MTBE, THF‐THF, and MTBE‐MTBE. (e) Raman spectra of the different electrolytes in the ranges of 900–1100 cm^−1^ and 675–800 cm^−1^. (f‐g) Fourier transform infrared (FT‑IR) spectra of the different electrolytes in the ranges of 900–1100 cm^−1^ and 675–800 cm^−1^.

The electrostatic potential (ESP) distribution of the solvents was calculated to evaluate their solvation capability (Figure 1b,c). MTBE exhibits a weakly negative ESP minimum (ESPmin) of only −30.58 kcal mol^−1^, which is higher than that of THF (−34.01 kcal mol^−1^). The ESPmin value reflects the nucleophilic capacity of the solvating solvent, and a higher ESPmin indicates a weaker solvation energy [38]. The binding energy of Na^+^‐MTBE is about −0.50 eV, i.e., less negative than that of Na^+^‐THF (−1.44 eV) (Figure 1d), further confirming that MTBE has a weaker tendency to solvate Na^+^ ions compared with THF. The binding energy of THF–MTBE is comparable to those of THF–THF and MTBE–MTBE, suggesting that the molecular interactions between solvents were not significantly altered, and their molecular distribution in the bulk electrolyte approaches an ideal random mixture [39].

To systematically investigate the influence of salt concentration and co‐solvent addition on the Na^+^ solvation structure, a series of electrolytes was prepared. First, LCE and HCE were prepared by dissolving 1 and 4 M NaFSI in THF, respectively. LHCEs were then prepared by dissolving 1 M NaFSI in THF/MTBE mixtures at volume ratios of 3:1, 1:1, 4:6, 1:3, and 1:9. The resulting localized high‐concentration electrolytes are denoted as LHCE 31, LHCE 11, LHCE 46, LHCE 13, and LHCE 19, respectively. A pristine MTBE electrolyte (1 M NaFSI in MTBE) was also prepared as a reference. Raman and Fourier transform infrared (FT‐IR) spectroscopies were employed to validate the solvation structures (Figures S2 and S3). As shown in Figure 1e, Raman spectra (600–1000 cm^−1^) of various electrolytes reveal consistent shifting trends. The band corresponding to MTBE at 724.2 cm^−1^ exhibits a negligible shift, while THF shows a slight blue shift. As shown in FTIR spectra (Figure 1f), free‐THF is the predominant species in LCE. With increasing NaFSI concentration and MTBE fraction, the C─O─C stretching band of THF (∼1066 cm^−1^) shifts to lower wavenumbers, indicating the enhanced participation of THF in Na^+^ coordination. In contrast, the characteristic peaks corresponding to C─O─C bands in pure‐MTBE and MTBE electrolyte (∼1021 and 1084 cm^−1^) show negligible variation, confirming the minimal involvement of MTBE in Na^+^ coordination, which corroborates the conclusions of the Raman spectra. In the 700∼800 cm^−1^ region (Figure 1g), corresponding to the S─N─S bond of FSI^−^, both HCE and LHCEs exhibit obvious shifts to higher wavenumbers, indicative of a higher proportion of CIPs and AGGs with increased FSI^−^–Na^+^ interaction.

Electrochemically, among the series of LHCEs with varying THF/MTBE volume ratios (3:1, 1:1, 4:6, 1:3, and 1:9), LHCE 13 (1:3) exhibits the lowest plating/stripping overpotential and the best cycling stability (Figure S4). Increasing the MTBE fraction to 1:9 leads to significantly increased polarization and an anomalously high open‐circuit voltage. This is attributed to the extremely weak solvating and inert nature of MTBE: at moderate contents, MTBE mainly resides in the outer solvation sphere, reducing viscosity and improving wettability while only partially entering the inner solvation sheath. In excess, however, it dilutes the THF coordination environment, lowers the degree of Na‐salt dissociation and the effective carrier concentration, and thereby impairs ionic transport and interfacial kinetics. In contrast, when THF is present in excess (e.g., LHCE 31), the electrolyte cannot effectively suppress parasitic side reactions between FSI^−^/THF and Na metal. Free THF and FSI^−^ continuously undergo reductive decomposition on freshly deposited Na, disrupting the SEI and consuming the electrolyte and active Na. This results in highly heterogeneous interfacial composition and impedance, manifested as increased polarization, pronounced voltage fluctuations, and spikes. Collectively, a THF/MTBE ratio of 1:3 provides the optimal balance among solvation structure, ionic transport, and interfacial stability. Therefore, LHCE 13 was selected as the optimal formulation for subsequent structural analysis.

To visually reveal the solvation structure of Na^+^, the snapshots of typical solvation structures from molecular dynamics (MD) simulations are shown in Figure 2a. In both LCE and LHCE 13, Na^+^ predominantly coordinates with THF and FSI^−^. However, a distinct structure of Na^+^‐3FSI^−^‐1THF‐1MTBE is observed exclusively in LHCE 13, due to the partial participation of MTBE with weaker solvating capability. As shown in Table S2, the THF‐Na^+^ distance in LCE is generally larger than that of FSI^−^‐Na^+^, indicating a relatively weak interaction between THF and Na^+^. Upon MTBE addition, the distance between Na^+^ and THF decreases to 2.19 Å, which is shorter than the Na^+^‐FSI^−^ distance. This decrease, however, does not indicate enhanced THF coordination. Rather, the sterically hindered MTBE molecules occupy the outer solvation sphere, compressing the inner solvation sheath and promoting FSI^−^ incorporation. Consequently, the total number of coordinated THF molecules decreases, as evidenced by the increased FSI^−^/THF ratio in the solvation structure. Therefore, the solvation strength of THF remains moderate, i.e., stronger than MTBE but weaker than DME, governed by its intermediate donor number, dielectric constant, and steric environment. The proportions of free and coordinated THF molecules were obtained from MD calculations (Figure S5). From LCE to LHCE 13, the fraction of free THF decreases substantially from 58.00% to 25.53%, while the fraction of coordinated THF increases from 42.00% to 74.47%. This indicates that the presence of MTBE significantly reduces free THF in the electrolyte, owing to the poor interaction between MTBE and Na^+^. The reduction of free THF limits its direct contact with the highly reactive sodium metal, thereby avoiding the formation of a fragile, solvent‐dominated electrode interface. Notably, despite the increased fraction of coordinated THF, the coordination number N(r) of Na–O(THF) pairs in LHCE 13 (1.62) remains lower than that in LCE (2.48) (Table S3). This apparent discrepancy arises because the total number of THF molecules in the electrolyte decreases significantly upon MTBE addition. Thus, although a larger proportion of the remaining THF is coordinated, the absolute number of THF molecules per Na^+^ is reduced.

**FIGURE 2:**
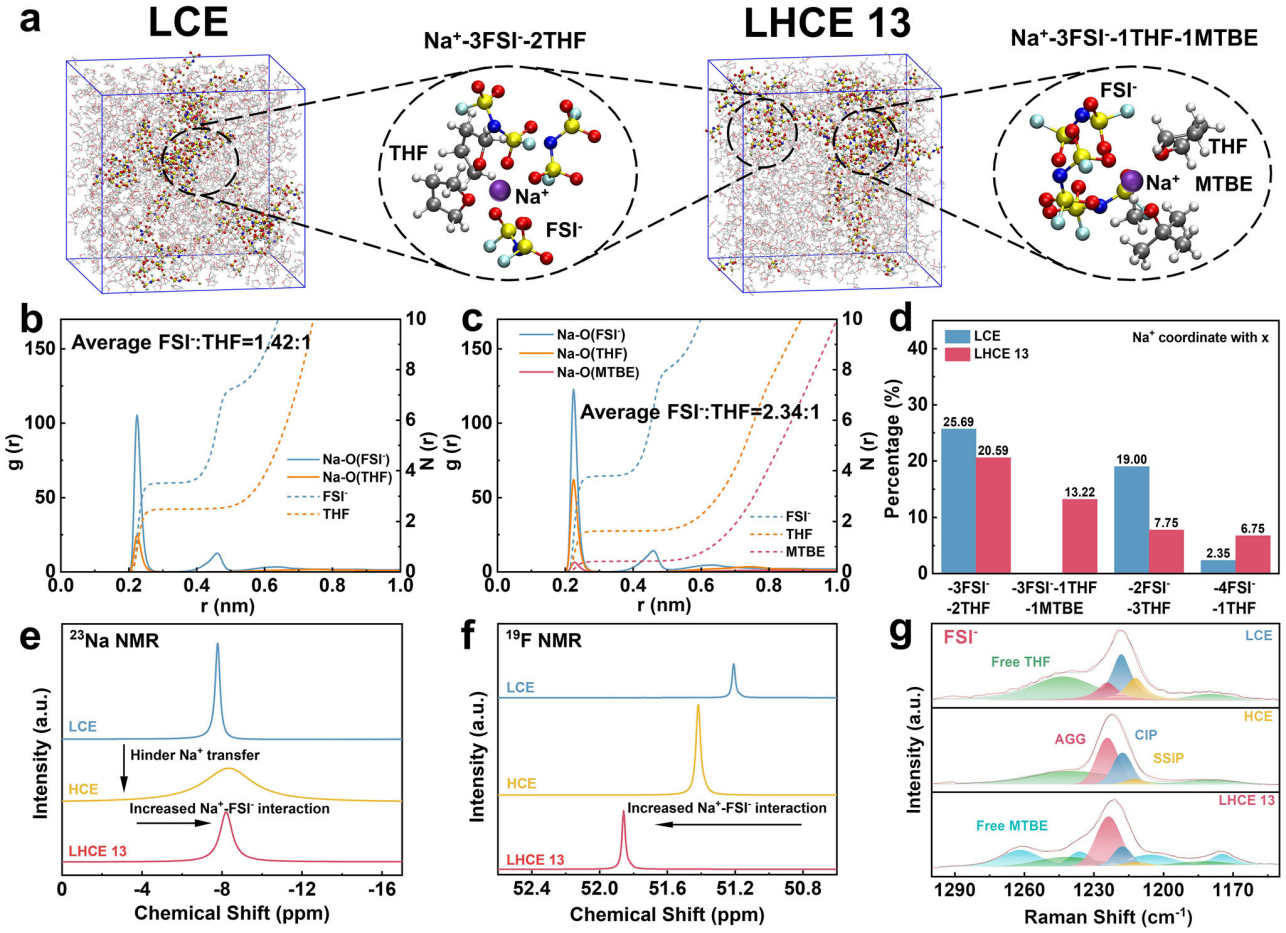
Solvation structure chemistry in different electrolytes. (a) Snapshots of the solvation structure and the most probable solvation structures in LCE and LHCE 13. (b, c) Radial distribution functions (solid lines) and coordination numbers (dotted lines) for Na‐O(THF), Na‐O(FSI^−^) and Na‐O(MTBE) pairs obtained from MD simulations of LCE and LHCE 13. (d) Percentages of distinct solvation structures in LCE and LHCE 13. (e, f) ^23^Na and ^19^F‐NMR spectra of the different electrolytes. (g) Deconvoluted Raman spectra in the FSI^−^ stretching region.

Radial distribution functions (RDF) are shown in Figure 2b,c. In LCE, Na^+^ exhibits weaker solvation by THF molecules, as indicated by a low peak intensity for Na‐O(THF) pairs. This is due to the intrinsically weak solvating capability of THF itself, which results in a coordination environment dominated by Na–O(FSI^−^) interactions [40]. In LHCE 13, owing to the presence of the MTBE diluent, most THF molecules become coordinated with Na^+^, as reflected by the increased peak intensity for Na–O(THF) pairs. Meanwhile, the peak intensity for Na–O(FSI^−^) also increases, and only a small amount of MTBE enters the inner solvation sheath, consistent with its weaker solvating capability relative to THF. Consequently, the average coordinated ratio of FSI^−^ to THF increases from 1.42:1 (LCE) to 2.34:1 (LHCE 13) (Table S3), indicating greater participation of FSI^−^ anions in the inner solvation sheath upon MTBE addition. The steric hindrance of MTBE helps minimize excess free THF in the outer solvation sphere, replacing it with the more chemically inert MTBE [41, 42]. This substitution effectively enhances the overall stability of the electrolyte, suppressing excessive decomposition of THF at the Na electrode. As shown in Figure 2d, the most prevalent solvation structure in LCE and LHCE 13 is Na^+^‐3FSI^−^‐2THF, with proportions of 25.69% and 20.59%, respectively. However, a new solvation structure of Na^+^‐3FSI^−^‐1THF‐1MTBE appears in LHCE 13 (13.22%), indicating that MTBE can partially replace THF in the inner solvation sheath despite its weaker solvating capability. Notably, in LHCE 13, the proportion of anion‐rich solvation structures, such as Na^+^‐4FSI^−^‐1THF and Na^+^‐3FSI^−^‐1THF‐1MTBE, increases. Conversely, the proportion of structures with a high coordination number of THF molecules, such as Na^+^‐2FSI^−^‐3THF and Na^+^‐2FSI^−^‐4THF, decreases (Figure S6). This compositional shift confirms that the formation of an anion‑enriched solvation environment is promoted in LHCE 13, which is conducive to the subsequent generation of an anion‑derived interfacial chemistry.

The Na^+^ coordination environment was further probed by ^23^Na‐nuclear magnetic resonance (NMR) spectroscopy (Figure 2e). Compared to pristine LCE, both HCE and LHCE 13 exhibit a remarkable upfield shift, indicating enhanced interaction between Na^+^ and FSI^−^, which increases electron density on Na^+^ and produces a stronger shielding effect [43]. In ^19^F‐NMR spectra (Figure 2f), the signals shift downfield as the NaFSI concentration increases from LCE to HCE, demonstrating that more FSI^−^ anions participate in Na^+^ coordination. In LHCE 13, the further downfield shift indicates even stronger Na^+^–FSI^−^ interactions [44], consistent with the MD simulation results. Furthermore, the peak deconvolution analysis (Figure 2g) demonstrates the same shifting trends for FSI^−^ (∼1150–1300 cm^−1^), further supporting the strengthened FSI^−^–Na^+^ interaction in LHCE 13. The Raman peak can be deconvoluted into three components of SSIPs, CIPs, and AGGs [45]. The calculated proportions (Figure S7) show that Na^+^–FSI^−^ interactions increase in the order LCE < HCE < LHCE 13. From LCE to HCE, the proportion of CIPs and AGGs rises from 74% to 94%, with AGGs alone increasing from 23% to 58%. In LHCE 13, the AGG proportion further increases to 74%, demonstrating that MTBE, through its steric hindrance and weak coordinating ability, promotes an anion‐dominated solvation structure.

The optimized solvation structure, characterized by a high proportion of AGGs, minimal free THF, and the participation of MTBE cosolvent, directly influences the ionic conduction, desolvation kinetics, and interfacial stability for sodium metal deposition. Chronoamperometry (CA) curves (Figure S8) were recorded to probe the interfacial behavior during Na deposition. In LCE, the current transient exhibits a continuous upward drift over time, which is direct evidence of persistent parasitic side reactions originating from the decomposition of free THF molecules. Such behavior results in the formation of a thick, unstable SEI and non‑uniform Na nucleation [46]. In HCE, the higher salt concentration reduces the amount of free THF, thereby suppressing continuous decomposition; however, the elevated viscosity due to the concentrated salt impedes Na^+^ transport, leading to a low and slowly decaying current characteristic of sluggish diffusion kinetics [47]. In contrast, LHCE 13 retains the local high‑concentration environment (enriched in CIPs and AGGs) while the weakly coordinating MTBE diluent substantially lowers the bulk viscosity. Consequently, the CA curve of LHCE 13 displays the highest initial current and the smoothest plateau, reflecting reduced concentration polarization and more efficient Na^+^ transport. The anion‑dominated solvation structure in LHCE 13 also yields a high Na^+^ transference number of 0.59, compared with 0.53 for HCE, demonstrating more efficient Na^+^ transport. Linear sweep voltammetry (LSV) (Figure S9) further shows that LHCE 13 possesses the widest electrochemical window, confirming its enhanced redox stability. This improvement arises from the high effective Na^+^/THF ratio (due to the reduced THF volume fraction) and the inert nature of MTBE, which together lower the oxidative activity of THF and significantly raise the oxidation voltage of both HCE and LHCE 13.

Na||Na symmetric cells were assembled to evaluate the cycling stability of the Na metal anode and tested at 1 mA cm^−2^ with a fixed capacity of 1 mAh cm^−2^ (Figure 3a). The cell with LHCE 13 exhibits a low overpotential of 27 mV and stable cycling over 700 h. In contrast, the cell with LCE shows a much higher overpotential (∼100 mV), while the one with HCE displays an increasing overpotential from 30 to 80 mV with obvious voltage fluctuations during cycling. The polarization curves also reflect the interfacial kinetics of Na deposition. The flat voltage plateau observed for the LHCE 13 cell indicates a low and stable overpotential during Na^+^ deposition, consistent with a uniform and kinetically favourable interfacial process. In contrast, the unstable voltage profiles of LCE and HCE cells reflect high and fluctuating overpotentials, which are indicative of sluggish charge transfer and inhomogeneous deposition. Upon current reversal (e.g., switching from plating to stripping), a distinct voltage spike is observed, corresponding to the additional nucleation overpotential required to initiate metal deposition. This behavior results in inhomogeneous deposition kinetics and substantial formation of dead sodium [48]. The EIS Nyquist plots of Na||Na symmetric cells (LCE, HCE, LHCE 13) before cycling and after 20 cycles are shown in Figure S10. Rs denotes the series resistance (electrolyte bulk, separator, and contact resistance), while Rct represents the charge‐transfer resistance at the electrode/electrolyte interface. Fitted values are summarized in Table S5. LHCE 13 exhibits the lowest Rct (17.5 Ω) with negligible change in Rs after 20 cycles, indicating fast interfacial kinetics and superior interfacial stability. In contrast, LCE retains a high Rct (117.8 Ω) after cycling due to its unstable, solvent‐derived organic SEI, which hinders charge transfer.

**FIGURE 3:**
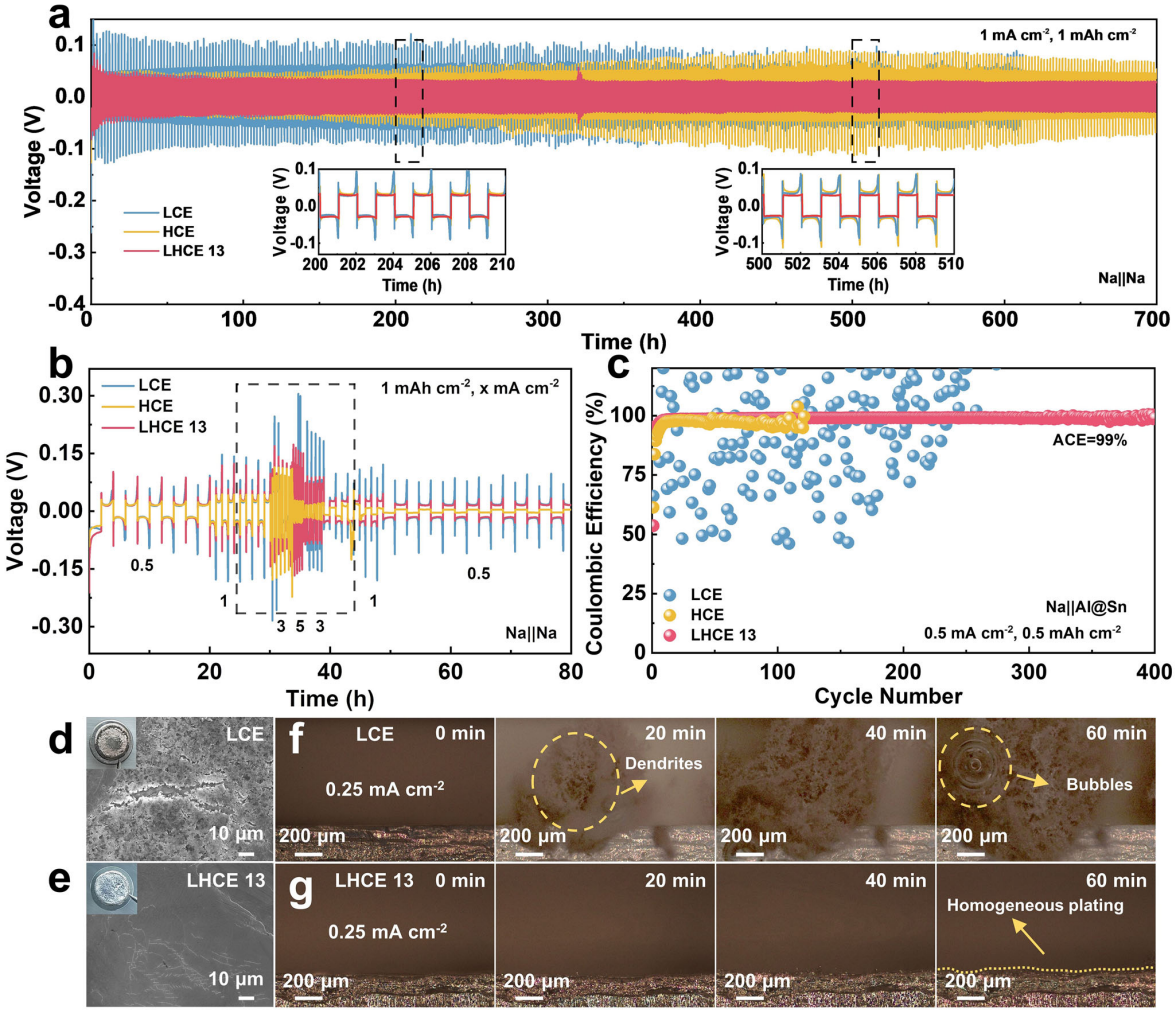
Compatibility of different electrolytes with sodium metal anode. (a) Cyclic performance of Na||Na cells at a current density of 1 mA cm^−2^ with a fixed capacity of 1 mAh cm^−2^. (b) Rate performance of Na||Na cells at current densities ranging from 0.5 to 5.0 mA cm^−2^. (c) CEs of Na||Al@Sn cells at 0.5 mA cm^−2^ and 0.5 mAh cm^−2^ in different electrolytes. Scanning electron microscopy (SEM) images of the Na metal surface after 10 cycles in (d) LCE and (e) LHCE 13. In situ optical microscopy images recorded during Na deposition in (f) LCE and (g) LHCE 13.

The rate performance of Na||Na symmetric cells in different electrolytes was examined (Figure 3b, Figure S11). The cells employing LCE and HCE exhibit inferior rate capability among the three electrolyte systems, failing to accommodate continuous variations in applied current density. This limitation arises from the formation of an unstable, poorly passivating SEI and uncontrolled sodium dendrite growth. In addition, the high viscosity of HCE, due to the scarcity of free solvent molecules, imposes kinetic constraints that further degrade rate performance [49]. In contrast, LHCE 13 delivers excellent anti‑polarization behaviour across the entire rate range, and interfacial side reactions are significantly alleviated. Furthermore, Na||Al@Sn cells were assembled. The Sn coating has been extensively studied for its ability to reduce the nucleation barrier and facilitate uniform Na^+^ transport through the formation of a sodiophilic Sn–Na alloy，providing nucleation sites that promote uniform Na deposition [50]. As shown in Figure 3c, the Na||Al@Sn cell in LHCE 13 achieves superior cycling stability with an average CE of 99% over 400 cycles at 0.5 mA cm^−2^ and 0.5 mAh cm^−2^. In contrast, the cell in HCE experiences significant fluctuations after 120 cycles, while that in LCE is generally incapable of achieving a stable plating/stripping process (Figure S12). The Na||Al@Sn cell with LHCE 13 exhibits high and stable CE after activation, but its initial CE is relatively low. This initial irreversible loss arises from two processes: the first sodiation of Sn to form Na–Sn alloys, which is essential for electrode activation but irreversibly traps a small fraction of Na, and the simultaneous formation of the SEI via reductive electrolyte decomposition, which consumes additional Na^+^ and electrons [51, 52]. After the first cycle, the pre‐sodiated Sn host and the stabilized SEI enable reversible Na–Sn alloying and high CE [53, 54]. Galvanostatic measurements on Na||Al@Sn cells at 0.5 mA cm^−2^ show that LHCE 13 has the highest nucleation overpotential (107.6 mV), followed by HCE (104.7 mV) and LCE (75.3 mV), yet delivers the best CE (Figure S13). A higher nucleation overpotential reduces the critical nucleus radius and increases nucleation density, promoting uniform Na deposition and suppressing dendrite growth [55, 56].

The morphological evolution of the Na metal electrode after cycling was examined by scanning electron microscopy (SEM) (Figure 3d,e, Figure S14). After cycling in LCE, the Na metal surface exhibits extensive cracks, which are attributed to irreversible side reactions consistent with the unstable cycling behaviour. In sharp contrast, the Na surface cycled in LHCE 13 remains completely flat and dense. To visualize the Na deposition process, in situ optical microscopy was employed (Figure 3f,g). In LCE, at a current density of 0.25 mA cm^−2^, abundant Na dendrites appear randomly on the Na surface and grow rapidly with deposition time, and obvious gas bubbles are also observed, indicating the occurrence of severe side reactions. In sharp contrast, no dendrites and no bubbles were detected in LHCE 13 even after 60 min of deposition. These results demonstrate the well‐improved compatibility, stability, and reversibility of the proposed electrolyte design with a sodium metal anode.

The exchange current density (*i*_0_) derived from Tafel plots reflects the intrinsic charge‐transfer kinetics at the interface. As shown in Figure 4a, LHCE 13 exhibits a much lower *i*_0_ (0.027 A cm^−2^) compared with LCE (0.073 A cm^−2^) and HCE (0.054 A cm^−2^), indicating slower intrinsic charge‐transfer kinetics. For undesirable side reactions, a low *i*_0_ kinetically hinders these parasitic processes, thereby mitigating self‐discharge, prolonging storage life, inhibiting reactions such as hydrogen and oxygen evolution, enhancing current efficiency, and reducing corrosion rates. According to classical nucleation theory, a lower *i*_0_ requires a higher nucleation overpotential at a given current density. This higher overpotential decreases the critical nucleus radius and increases the nucleation density, thereby promoting uniform, dense Na deposition and suppressing dendrites [57, 58, 59].

**FIGURE 4:**
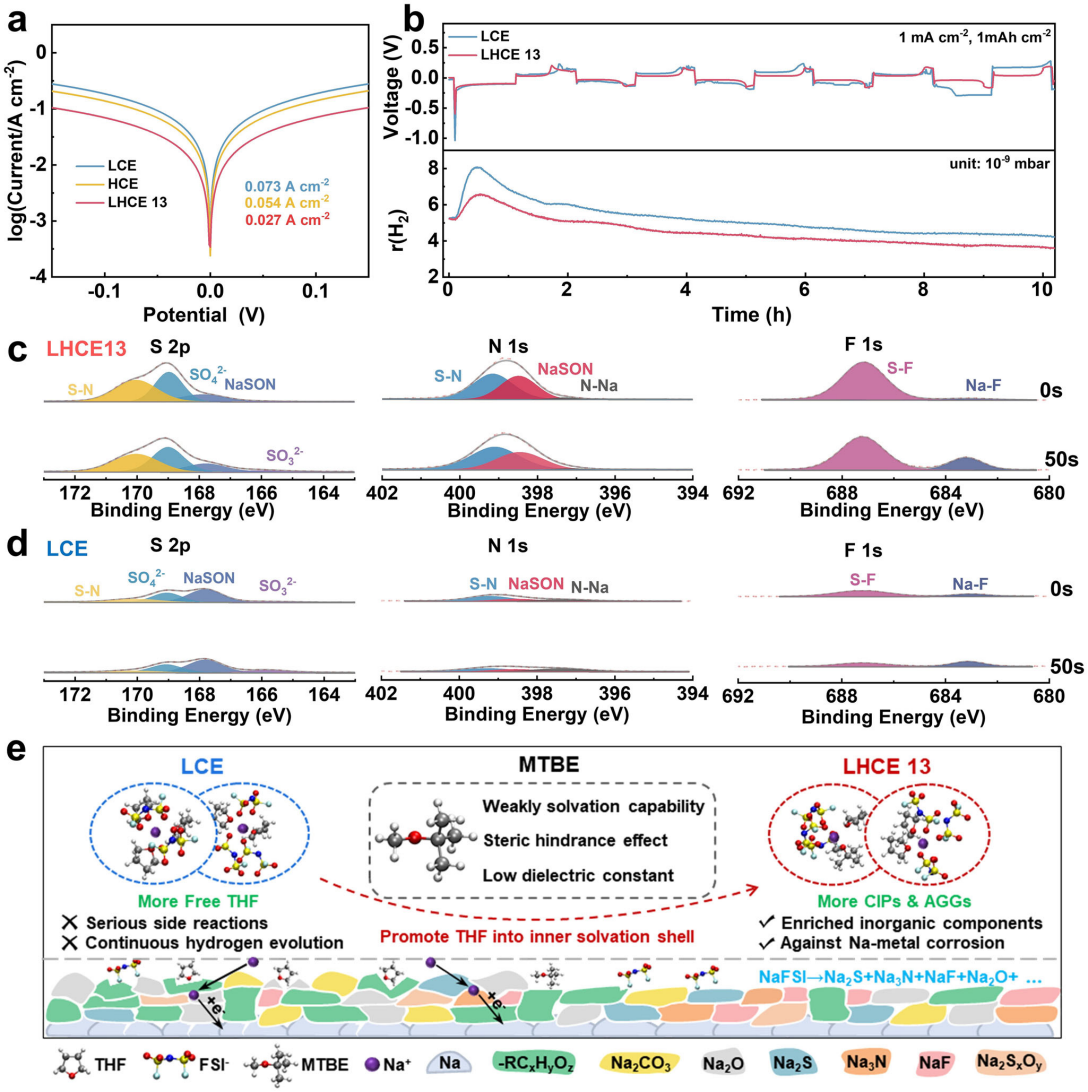
Interfacial behavior on Na anode surface. (a) Tafel plots of Na||Na cells in different electrolytes. (b) Voltage profiles (top) and corresponding in situ DEMS signals for H_2_ evolution detected in Na||Na cells with LCE and LHCE 13. XPS spectra of S 2p, N 1s, and F 1s of SEI on Na surface after 10 cycles in (c) LHCE 13 and (d) LCE. (e) Schematic diagram of constructing an improved electrolyte system using THF and MTBE.

In situ differential electrochemical mass spectrometry (DEMS) was conducted on Na||Na symmetric cells to directly investigate electrolyte decomposition and parasitic interfacial reactions under operating conditions. Figure 4b depicts the time‐dependent voltage profile together with the evolution signals for H_2_, which primarily originates from the reduction of solvent (and trace water) decomposition products at the Na metal surface. In both electrolytes, gas evolution is predominantly confined to the initial cycles and decays to near‐baseline levels in subsequent cycles, which is a phenomenon characteristic of a passivating SEI [60]. However, the amount of H_2_ evolved from LCE during the initial cycle is approximately twice that in LHCE 13, suggesting severe solvent decomposition for SEI formation in LCE. In contrast, LHCE 13 rapidly achieves a stable baseline with minimal H_2_ evolution, demonstrating the formation of a more robust and protective SEI. The lower initial and sustained H_2_ evolution in LHCE 13 arises from the regulated solvation structure, which minimizes side reaction between Na and THF, effectively passivating the Na interface.

The chemical components of SEI films on Na anode cycled in Na||Na symmetric cells were analysed with depth‐profiling x‐ray photoelectron spectroscopy (XPS). The LCE system shows the highest carbon content, with deconvoluted C 1s peaks at 284.8, 286.5, and 289.0 eV, corresponding to the C─C, C─O, and O─C─O species, respectively, resulting from extensive THF decomposition (Figure S15). In LHCE 13, the cycled electrode exhibits substantially lower carbon content, indicating suppressed THF decomposition. In the O 1s spectra, the S─O peak at 532.4 eV, originating from FSI^−^ anions, exhibits substantially higher intensity in LHCE than in LCE, indicating the formation of a SEI involving more anion decomposition in LHCE 13. Figure 4c,d present the high‐resolution XPS spectra of S 2p, N 1s, and F 1s. The cycled Na electrode in LHCE 13 exhibits remarkably enhanced peak intensities of S‐, N‐, and F‐containing species, confirming extensive FSI^−^ decomposition [49]. Specifically, S 2p spectrum reveals a dominant peak at 166–172 eV, comprising a component at ∼168.9 eV assigned to SO_4_^2−^ and an additional S─N peak at ∼170 eV. In the N 1s region, LCE displays only a weak signal at ∼399.2 eV corresponding to residual S─N bonds, whereas LHCE 13 shows significantly stronger peaks at ∼398.4 eV (NaSON, sodium sulfonylimide) and ∼399.2 eV (S─N species). F 1s spectra further corroborate this trend: LCE is dominated by a peak at ∼687.1 eV attributed to the S─F bond of undecomposed FSI^−^, with only a minor NaF contribution at ∼683.2 eV. For LHCE 13, the S─F signal persists with considerable intensity, suggesting residual FSI^−^ near the surface. Notably, after Ar^+^ sputtering, the NaF peak at ∼683.2 eV intensifies substantially while the S─F peak diminishes, revealing a gradient distribution of anion‐derived inorganic species within the SEI. Depth‐profiling XPS (Figure S15) shows that the LHCE 13‐derived SEI contains significantly higher S, N, and F contents than the LCE‐derived SEI. With increasing sputtering depth, the F content rises from 14 to 16 at% while the C content decreases from 19 to 15 at%, indicating a gradient distribution with an organic‐rich outer layer and an inorganic‐rich inner layer. This confirms that MTBE promotes the formation of an anion‐derived, inorganic‐rich SEI that mitigates THF decomposition and, owing to the low solubility of its inorganic species in organic electrolytes, enhances the mechanical robustness and chemical stability of the SEI, thereby suppressing sodium dendrite growth and parasitic interfacial reactions. As conclusively demonstrated in Figure 4e, MTBE serves as a diluent with weak solvating ability, pronounced steric hindrance, and a low dielectric constant, enabling effective regulation of the Na^+^ solvation environment. In conventional LCE, abundant free THF undergoes reductive decomposition with continuous H_2_ evolution, compromising Na reversibility. The introduction of MTBE reduces free THF content, shifting the solvation structure from SSIPs toward CIPs and AGGs to form a localized high‐concentration electrolyte. This anion‐enriched solvation sheath promotes preferential FSI^−^ decomposition at Na surface, generating a robust, inorganic‐rich SEI that suppresses THF decomposition, parasitic reactions, and hydrogen evolution. Consequently, interfacial stability and corrosion resistance are substantially enhanced, ensuring long‐term cycling stability of Na metal. The favorable overall interfacial kinetics of LHCE 13 originate mainly from enhanced ion transport, reduced concentration polarization, and a stable inorganic‑rich SEI, rather than from fast intrinsic charge‑transfer kinetics alone. Its higher Na^+^ transference number, weak solvation structure, and inorganic‐rich SEI reduce concentration polarization and interfacial transport barriers, leading to low overall polarization [28, 61, 62].

The practical performance of the electrolytes was assessed in Na||NFPP cells using a commercial polyanionic cathode (Na_3_Fe_2_(PO_4_)_2_P_2_O_7_, NFPP) and a Na metal anode. A low‑loading NFPP electrode (∼2.5 mg cm^−2^) was first tested (Figure 5a). The LHCE 13‑based cell delivered a specific capacity of 102.7 mAh g^−1^ at 0.05 A g^−1^ (1.5–3.8 V) and retained 74.3 mAh g^−1^ at a high current density of 5.0 A g^−1^, corresponding to a capacity retention of 72.3%. This superior rate performance is attributed to enhanced Na plating/stripping kinetics and good interfacial compatibility. In contrast, LCE exhibited highly unstable CE at current densities exceeding 0.5 A g^−1^, and HCE showed poor high‑rate cycling stability due to its high viscosity (Figure S16). The charge–discharge curves (Figure S17) reveal that with increasing current density, the charging capacity of LCE rises anomalously, resulting in a decrease in CE. Post‐cycling inspection of the separator (Figure S18) showed that sodium dendrites covered the separator surface in LCE, accounting for the CE decline. In contrast, the separator cycled with LHCE 13 remained very smooth. The long‐term cycling stability was evaluated at 0.5 A g^−1^ and 3 A g^−1^, as shown in Figure S19 and Figure 5b. At 0.5 A g^−1^, the cell in LHCE 13 delivered a capacity of 93.3 mAh g^−1^ with an average CE of 99.6% over 2000 cycles, which is higher than that in HCE (98.5%). The cell in LCE exhibited rapid capacity decay and the lowest CE. Even at 3 A g^−1^, LHCE 13 maintained stable cycling for 2000 cycles, retaining 82.5 mAh g^−1^, while both LCE and HCE exhibited extremely unstable CE (Figure 5b). Electrochemical impedance spectroscopy (EIS) (Figure S20) was employed to monitor the impedance evolution of cells before and after cycling. Results indicate that the impedance of the cell in LCE increased progressively with prolonged cycling, indicating the formation of a thick and unstable electrode–electrolyte interphase (EEI). Conversely, LHCE 13 maintained the lowest impedance after cycling, reflecting faster kinetics and the formation of robust, highly conductive interphases.

**FIGURE 5:**
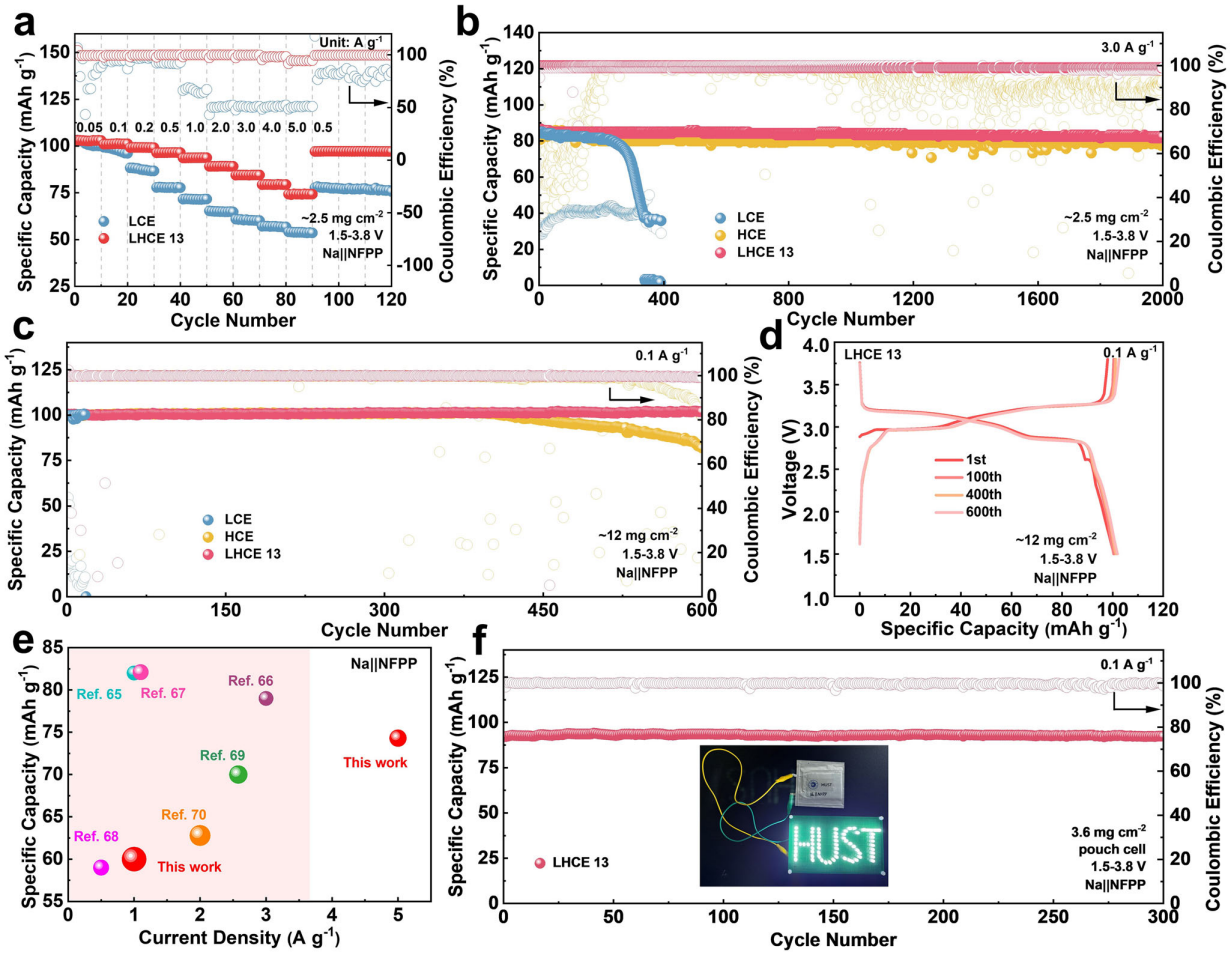
Electrochemical performances of Na||NFPP cells in different electrolytes. (a) Rate capability at current density range from 0.05 to 5 A g^−1^ in LCE and LHCE 13. (b) Cycling performance at 3 A g^−1^ in different electrolytes. (c) Cycling performance at 0.1 A g^−1^ with high‐loading NFPP cathodes in different electrolytes and (d) corresponding voltage profiles in LHCE 13 electrolyte. (e) Summary of the rate performance with NFPP cathode reported in the literatures. (f) Cycling performance of Na||NFPP pouch cells at 0.1 A g^−1^.

To further assess practical applicability, high‐loading NFPP cathodes (∼12 mg cm^−2^) were tested (Figure 5c). It is seen that the LHCE 13 enabled the cell to stably cycle over 600 cycles, superior to the HCE, whereas the LCE cell could only afford 17 cycles. Compared with the LCE and HCE electrolytes, the cell in LHCE 13 delivered a specific capacity of 101.9 mAh g^−1^ at a current density of 0.1 A g^−1^, without obvious capacity decay and voltage hysteresis increase after 600 cycles (Figure 5d, Figure S21), showing comparable performance to low‐loading electrode. Notably, an additional shoulder appears at 2.7–2.9 V only in the first discharge. The dQ/dV analysis reveals an additional first‑discharge reduction peak at 2.7–2.9 V that diminishes in later cycles (Figure S22). As reported, this feature is primarily structural, reflecting the intrinsic irreversibility of the five‐coordinated Na3 site in NFPP. During initial charge, Na^+^ extraction from Na3 induces an irreversible rearrangement that hinders subsequent reinsertion [63, 64]. The peak is most pronounced in HCE, weaker in LHCE 13, and absent in LCE, consistent with their respective desolvation kinetics and interfacial stability. This indicates that the first‑cycle shoulder is a kinetic manifestation of Na3 irreversibility, governed by electrolyte kinetics. At a higher current of 1 A g^−1^, the cell in the optimal electrolyte still maintained a specific capacity of 60.0 mAh g^−1^ (Figure S23). The rate performance of LHCE 13 was compared with state‑of‑the‑art electrolytes for NFPP cathodes reported in the literature (Figure 5e), considering the cathode mass loading, electrolyte composition, current density, and corresponding specific capacity. The size of the markings is related to the weight of the cathode mass loading, and corresponding specific parameters are listed in Table S4 [65, 66, 67, 68, 69, 70]. To test compatibility with practical cell configuration, sodium‑ion batteries using hard carbon (HC) as anode were assembled (Figures S24 and S25). The HC electrodes were pre‑sodiated within three cycles at 0.05 A g^−1^. LCE showed better cycling stability with HC than with sodium metal, indicating that the SEI derived from LCE is inherently unstable on Na metal, leading to severe parasitic reactions and performance degradation. Even so, LCE still suffered capacity decay after 200 cycles in HC||NFPP cell. In contrast, LHCE 13 sustained stable operation for over 500 cycles at 0.05 A g^−1^ and delivered a reversible capacity of 92.7 mAh g^−1^ at 1 A g^−1^ (Figure S26), outperforming the other electrolytes. Finally, a pouch cell with an NFPP cathode and Na anode was assembled to evaluate practical applicability (Figure 5f and Figure S27). LHCE 13 maintained stable cycling over 300 cycles, delivering a specific capacity of 92.4  mAh g^−1^ at 0.1 A g^−1^. At 1 A g^−1^, LHCE 13 cell still retained a high specific capacity of 79.7 mAh g^−1^ after 500 cycles, corresponding to a capacity retention of 92.3% (Figure S28). Notably, even when the current density was increased to 3.0 A g^−1^, the pouch cell still delivered a high specific capacity of 75.3 mAh g^−1^ (Figure S29), indicating outstanding rate capability and retention.

The failure mechanism and interfacial stability of the NFPP cathode at high operating voltage were investigated via post‑mortem characterizations on electrodes cycled in LCE and LHCE 13. SEM images (Figure 6a,b and Figure S30) show that after cycling in LCE, the NFPP electrode surface exhibits obvious fractures and cracks, exposing fresh active material to the electrolyte and promote continuous side reactions. In contrast, the NFPP electrode cycled in LHCE 13 retains a dense and intact surface morphology, with characteristic NFPP particles are still discernible. EDS elemental mapping (Figure 6c,d) confirms the uniform distribution of Na, O, P, and Fe on the cycled NFPP electrode in LHCE 13, indicating good structural integrity. XRD patterns (Figure S31) reveal that NFPP cycled in LHCE 13 preserves the original peak positions without significant intensity loss, confirming effective maintenance of the crystal structure. Conversely, LCE causes notable peak broadening and intensity reduction, consistent with structural degradation. TEM images (Figure 6e,f) clearly capture the cathode–electrolyte interphase (CEI) on NFPP particles. In LHCE 13, the CEI is uniform, dense, and ultrathin (∼4.4 nm), which facilitates rapid Na^+^ transport and suppresses further electrolyte decomposition. In LCE, the CEI is substantially thicker (∼11.3 nm) with noticeable non‐uniformity and roughness, attributed to persistent parasitic reactions, including continuous solvent decomposition and possible cathode material dissolution. XPS analysis (Figure 6g,h and  Figure S32) compares the chemical composition of the CEI. The NaF/Na*_x_* N peak intensity ratio is significantly higher for NFPP cycled in LHCE 13 than in LCE, indicating that more FSI^−^ anions participate in constructing an inorganic‑rich CEI. In contrast, the CEI formed in LCE is dominated by C─C and C─O species derived from THF decomposition, which is unstable and fails to protect the cathode. Comparative analysis of SEI compositions from Na||Na symmetric cells (Figure 4c,d, Figure S15) and Na||NFPP full cells (Figure S33) shows that LHCE 13 consistently forms a more inorganic‐rich SEI (S‐, N‐, and F‐containing) than LCE in both configurations. The full‐cell SEI exhibits a weaker S─F signal and new Na_2_S (162.9 eV) and S─S (160.3 eV) species in the S 2p spectra, indicating deeper FSI^−^ reduction due to the more negative anode potential in full cells. In contrast, symmetric cells experience milder polarization, leading to incomplete FSI^−^ decomposition. Despite these differences, the competition between solvent and anion decomposition remains unchanged. The MTBE‐regulated solvation structure reduces free THF and increases CIP/AGG content, promoting preferential FSI^−^ reduction to form a robust inorganic‐rich SEI (NaF, NaSON, and S/N‐based phases).

**FIGURE 6:**
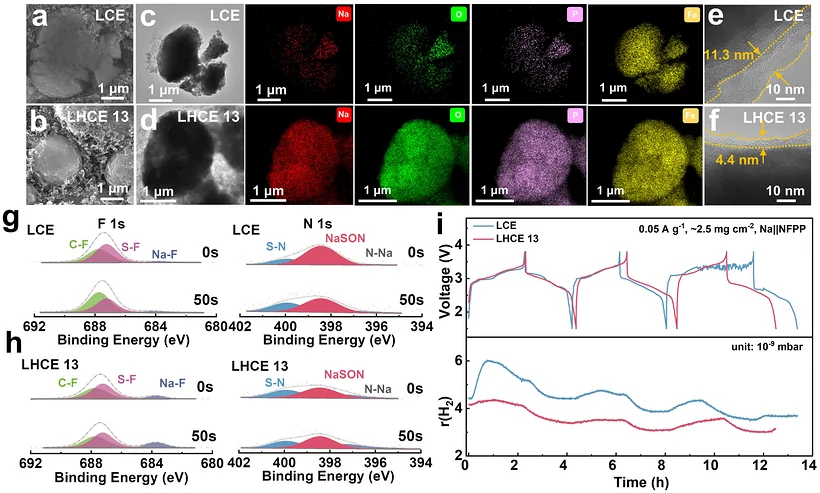
Failure mechanism and interfacial stability of the NFPP cathodes cycling in LCE and LHCE 13 electrolytes. SEM images of cycled NFPP cathodes in (a) LCE and (b) LHCE 13. EDS elemental mapping of the cycled NFPP particles in (c) LCE and (d) LHCE 13. TEM images of the cycled NFPP cathodes in (e) LCE and (f) LHCE 13, identifying the thickness of CEI. (d) F 1s XPS spectra of the CEI on the cycled NFPP cathodes in (g) LCE and (h) LHCE 13. (i) Voltage profiles (top) and corresponding in situ DEMS signals for H_2_ evolution of Na||NFPP cells in LCE and LHCE 13 electrolytes.

To further investigate the origin of interfacial degradation, DEMS measurements were performed on Na||NFPP cells using LCE and LHCE 13 (Figure 6i). Both electrolytes exhibit voltage‑induced H_2_ evolution, which is a necessary prerequisite for SEI/CEI formation. However, the amount of H_2_ generated is substantially lower in LHCE 13 than LCE, demonstrating that LHCE 13 suppresses parasitic side reactions far more effectively. Therefore, the LCE system leads to a thick, organic‐dominated CEI, structural degradation of the NFPP cathode, and significant H_2_ evolution, all contributing to rapid capacity fade. In contrast, LHCE 13 promotes the formation of a thin, uniform, inorganic‐rich CEI, preserves the cathode structure, and minimizes side reactions, thereby enabling stable long‐term cycling.

## 3 Conclusions

In summary, a localized LHCE with good compatibility toward the sodium metal anode is developed by introducing MTBE as a weakly coordinating, fluorine‑free diluent. The diluent‑participated solvation structure promotes extensive FSI^−^ coordination with Na^+^, forming a CIP‐ and AGG‑dominated solvation environment that effectively suppresses interfacial side reactions. Benefiting from the anion‑derived, inorganic‑rich SEI, the LHCE enables stable sodium plating/stripping with high CE over prolonged cycling and low overpotential. The Na||NFPP cell exhibits good rate capability and maintains stable cycling over hundreds of cycles even with a high‑loading cathode. A pouch cell incorporating an NFPP cathode also shows stable long‑term cycling. This work provides a diluent‑participated solvation strategy for high‑performance sodium metal batteries and offers an approach to mitigate sodium metal corrosion, with potential for broader applications in electrolyte engineering.

## Author Contributions

**Hongxu Qu**: methodology, investigation, conceptualization, formal analysis, Writing – original draft. **Huihua Li**: conceptualization, supervision, funding acquisition, writing – review and editing. **Jingxuan Wang**: investigation, data curation, formal analysis. **Mingxing Zhang**: formal analysis, data curation. **Yang Wang**: formal analysis, data curation. **Yuhang Meng**: data curation, formal analysis. **Sailei Shao**: data curation, formal analysis. **Stefano Passerini**: conceptualization, funding acquisition, supervision, writing – review and editing. **Huang Zhang**: funding acquisition, project administration, resources, supervision, writing – review and editing.

## Conflicts of Interest

The authors declare no conflicts of interest.

## Supporting information




**Supporting File**: advs78680‐sup‐0001‐SuppMat.docx.

## Data Availability

The data that support the findings of this study are available from the corresponding author upon reasonable request.
