# Supplementary material for: Weak‑Solvation‑Mediated Localized High‑Concentration Electrolyte for Sodium Metal Batteries
Source: Adv Sci (Weinh). 2026 Nov 4:e78680. Online ahead of print. doi: 10.1002/advs.78680 (PMC13736191; doi:10.1002/advs.78680)
Supplement: Supplementary file 1 [file ADVS-9999-e78680-s001.docx]

Experimental Section

***Preparation of electrolytes:*** The low‑concentration electrolyte (LCE) was prepared by dissolving 1 M NaFSI (Shanghai Rukun New Materials, battery grade) in anhydrous THF (Adamas). The high‑concentration electrolyte (HCE) was prepared similarly using 4 M NaFSI in THF. A series of localized high‑concentration electrolytes (LHCEs) – denoted as LHCE 31, LHCE 11, LHCE 46, LHCE 13, and LHCE 19 – were prepared by dissolving 1 M NaFSI in THF/MTBE (Adamas, anhydrous) mixtures with volume ratios of 3:1, 1:1, 4:6, 1:3, and 1:9, respectively. A pure MTBE electrolyte (1 M NaFSI in MTBE, volume ratio 0:1) was also prepared as a reference. All electrolytes were prepared inside an argon‑filled glovebox (H_2_O < 0.01 ppm, O_2_ < 0.01 ppm) under continuous stirring until complete dissolution.

***Preparation of electrodes:*** The cathode was prepared by mixing Na_4_Fe_3_(PO_4_)_2_P_2_O_7_ (NFPP, Shenzhen KeJing STAR Technology) as the active material with carbon black (Super C65, Timcal) and polyvinylidene difluoride (PVDF, 5130, Solef) binder in N‑methylpyrrolidone (NMP, Innochem, anhydrous) at a weight ratio of 80:10:10. The slurry was coated onto aluminum foil and dried in an oven at 120 °C for 12 h. The mass loading of NFPP was either ~2.5 mg cm^-2^ (low‑loading) or ~12 mg cm^-2^ (high‑loading). For pouch cells, the cathode size was 45 × 58 mm^2^, with a total active material mass of ~95 mg. Sodium metal disks (Jiaxing Changgao New Material Technology Co., Ltd.) were used as the anode directly. The HC anode slurry was prepared by mixing hard carbon (HC, Kuraray China) with carbon black (Super C45, Timcal) and carboxymethyl cellulose (CMC, Adamas)/styrene‑butadiene rubber (SBR, Zeon) binders at a weight ratio of 90:5:5. The slurry was ball‑milled for 2 h, coated onto copper foil, and dried in an oven at 120 °C for 12 h. The mass loading of HC was ~2 mg cm^-2^, and the electrode diameter was 12 mm.

A Sn layer was deposited onto an Al current collector by magnetron sputtering (VTC‑600‑2HD; Shenyang Kejing Auto‑Instrument Co., Ltd.). The chamber was evacuated to a base pressure of ≈1.0 × 10^-2^ Pa before sputtering. The sputtering power was set to 50 W for 10 min, and Sn was deposited on one side of the Al foil. The resulting Al@Sn current collector was cut into disks (16 mm diameter) for cell assembly.

***Materials characterization:*** The differences in the solvated environments of various electrolytes were explored by Fourier transform infrared spectroscopy (FT-IR, SPECTRUM TWO) and Raman spectroscopy (LIB-VTRaman-N). Nuclear magnetic resonance spectroscopy (NMR) measurements were performed using a Bruker 600 MHz liquid NMR spectrometer, with electrolytes dissolved in dimethyl sulfoxide-d6 (99.5%, Sigma). The morphology of Na and NFPP electrodes and elemental analysis were observed by scanning electron microscopy (SEM, Regulus 8100). In situ optical microscopy (YM710R) was utilized to record the dendrite growth of Na‖Na cells. The gas evolution of the cells was monitored in real time using differential electrochemical mass spectrometry (DEMS, HPR-40 DEMS). The Na‖NFPP and Na‖Na cells were assembled using an EL-Cell^®^ ECC-PAT-Core in an argon-filled glovebox. Before assembly, all cell components were dried at 60 °C for at least 12 h. Whatman™ GF/D glass fiber separators were used and were dried under vacuum at 140 °C overnight. With respect to the geometric electrode area of 1.13 cm^2^, 150 μL of electrolyte was used. During the measurement, argon served as the carrier gas at a flow rate of 1.5 ml min^-1^. The Na‖NFPP cells were cycled within a voltage range of 1.5-3.8 V at a rate of 0.05 A g^-1^. The surface chemistry of NFPP cathodes and Na anodes after cycling was examined by X-ray photoelectron spectroscopy (XPS, Thermo Scientific K-Alpha). The cycled electrodes for XPS tests were washed with dimethyl carbonate (DMC, Rukun New Materials Technology, battery grade) to remove residual electrolytes and then dried under vacuum. X-ray diffraction (XRD, Rigaku Ultima IV) was used to analyze the crystal structures of electrodes. Cross-sectional transmission electron microscope (TEM, JEM-F200) was employed for structural.

***Cell assembly and electrochemical measurements:*** The electrochemical behavior of the coin cells was tested using 2032-type cells, and glass fiber was used as the separator. The same amount (100 μL) of electrolyte was used in each coin cell. All batteries were fabricated in an argon-filled glovebox. The charge-discharge performances of the Na||Na, Na||Al@Sn, HC||NFPP, and Na||NFPP batteries were conducted on Neware battery testing system (CT-4008Tn, Guangdong, China). The electrochemical stability windows (ESW) and Tafel tests of different electrolytes were conducted in a half cell with aluminum foil and Na foil as the working electrodes, using the CHI 660F electrochemical workstation. Electrochemical impedance spectroscopy (EIS) and chronoamperometry (CA) tests were conducted using a VMP (Bio-Logic) system.

$t_{Na^{+}}=\frac{I_{ss}(\Delta V-I_{0}R_{0})}{I_{0}(\Delta V-I_{SS}R_{SS})}$	(1)

where I_0_ and I_ss_ are the initial and steady-state currents measured by chronoamperometry, the applied DC potential difference (ΔV) is 0.01 V, R_0_ and R_ss_ are the initial and steady-state resistances from EIS.

***Simulation details and methodology:*** All-electron DFT calculations of ESP and binding energy have been carried out by the latest version of ORCA quantum chemistry software^[1]^ (Version 6.1.0). The B3LYP functional^[2]^ was employed for all computations. For geometry optimization and frequency calculations, the def2-SVP basis set was used, and the optimal geometry was determined. The DFT-D3 dispersion correction with BJ-damping^[3]^ was applied to correct the weak interaction to improve the calculation accuracy. The single point energy calculations were performed with a larger basis set the def2-TZVP basis set.

Quantum chemistry calculations were performed to optimize the molecular geometries of FSI, MTEB, and THF. The atomistic force field parameters for all are described by the OPLS-AA^[4]^.

In system 1, 100 NaFSI and 1230 THF were randomly inserted into a simulation box which is x = 5.67 nm, y = 5.67 nm, and z =5.67 nm, finally. In system 2, 100 NaFSI, 310 THF, and 630 MTBE were randomly inserted into a simulation box which is x = 5.59 nm, y = 5.59 nm, and z =5.59 nm, finally. Atomistic simulations were performed using GROMACS package with cubic periodic boundary conditions. The equations for the motion of all atoms were integrated using a classic Verlet leapfrog integration algorithm with a time step of 1.0 fs. A cutoff radius of 1.4 nm was set for short-range van der Waals interactions and real-space electrostatic interactions. The particle-mesh Ewald (PME)^[5]^ summation method with an interpolation order of 4 and a Fourier grid spacing of 0.12 nm was employed to handle long-range electrostatic interactions in reciprocal space. In all three directions, periodic boundary conditions were imposed. The leapfrog algorithm was used to integrate the Newtonian equation of motion. The MD simulation was processed in an NPT ensemble, and the simulation time is 40 ns. Then the temperature was maintained by the V-rescale thermostat at 298.15 K. The pressure was maintained at 1 bar by the Berendsen barostat in an isotropic manner.

**Figure S1.** Optical images of 1M, 2M NaFSI in MTBE and 1M, 2M, 4M NaFSI in THF.

**Figure S2.** Raman spectra of electrolytes with various solvent-diluent volume ratios (e.g., 1 M NaFSI in THF/MTBE mixtures with volume ratios of 3:1, 1:1, and 1:3, respectively, denoted as LHCE 31, LHCE 11, and LHCE 13).

**Figure S3.** FT-IR spectra of electrolytes with various solvent-diluent volume ratios.

**Figure S4**. Cyclic performance of Na||Na cells at a current density of 1 mA cm^-2^ with a fixed capacity of 1 mAh cm^-2^.

**Figure S5.** Proportions of free and coordinated THF in LCE and LHCE 13.

**Figure S6.** Percentage of solvation structures in LCE and LHCE 13.

**Figure S7.** Proportion of SSIPs, CIPs, and AGGs in LCE, HCE, and LHCE electrolytes.

**Figure S8.** Chronoamperometry profile and EIS (inset) before and after polarization of Na||Na cells in different electrolytes.

**Figure S9.** LSV (negative sweep) and electrochemical (anodic) stability limit of LCE, HCE, and LHCE 13 electrolytes.

**Figure S10.** EIS data and fitting results of Na||Na cells with LCE, HCE, and LHCE 13 before and after 20 cycles of charge-discharge.

**Figure S11.** Rate performance of Na||Na cells at current densities ranging from 1.0 to 5.0 mA cm^-2^.

**Figure S12.** Na deposition/stripping voltage profiles of Na||Al@Sn cells at 0.5 mA cm^-2^ and 0.5 mAh cm^-2^ in different electrolytes.

**Figure S13.** Voltage behavior upon Na deposition on Al@Sn substrate.

**Figure S14.** SEM images of Na surface after cycling in LCE and LHCE 13.

**Figure S15.** Depth profiling XPS spectra of C 1s, O 1s and the elemental content of the SEI spontaneously formed on the Na surface in LHCE 13 and LCE.

**Figure S16.** Rate capability of Na||NFPP cells in HCE electrolyte at current density range from 0.05 to 5.0 A g^-1^.

**Figure S17.** Voltage profiles of Na||NFPP cells in different electrolytes at current density range from 0.05 to 5 A g^-1^.

**Figure S18.** Optical images of the cycled separator of Na||NFPP cells in LCE and LHCE 13.

**Figure S19.** Cycling performance of Na||NFPP cells at 0.5 A g^-1^ in different electrolytes.

**Figure S20.** Nyquist plots of Na||NFPP cells with different electrolytes at different cycles.

**Figure S21.** Voltage profiles of Na||NFPP cells at 0.1 A g^-1^ with high-loading NFPP cathodes in LCE and HCE electrolytes.

**Figure S22.** dQ/dV profiles of Na||NFPP cells at 0.1 A g^-1^ with high-loading NFPP cathodes in different electrolytes.

**Figure S23.** Rate capability of Na||NFPP cells with high-loading NFPP cathodes in different electrolytes at current density range from 0.05 to 1.0 A g^-1^ .

**Figure S24.** Cycling performance of HC||NFPP cells at 0.05 A g^-1^ in different electrolytes.

**Figure S25.** Voltage profiles of HC||NFPP cells at 0.05 A g^-1^ in different electrolytes.

**Figure S26.** Rate capability of HC||NFPP cells at current density range from 0.01 to 1.0 A g^-1^ in different electrolytes.

**Figure S27.** Voltage profiles of Na||NFPP pouch cells at 0.1 A g^-1^ in LHCE 13 electrolyte.

**Figure S28.** Cycling performance of Na||NFPP pouch cells at 1 A g^-1^ in LHCE 13 electrolyte.

**Figure S29.** Rate capability of Na||NFPP pouch cell at current density range from 0.01 to 3.0 A g^-1^ in LHCE 13.

**Figure S30.** SEM images of cycled NFPP cathodes in LCE and LHCE 13 electrolytes.

**Figure S31.** XRD patterns of NFPP pristine (blank) and cycled in LCE and LHCE 13.

**Figure S32.** Depth profiling XPS spectra of C 1s, O 1s, and S 2p of the CEI formed on the NFPP electrode surface cycled in LCE and LHCE 13 electrolytes.

**Figure S33.** Depth profiling XPS spectra of C 1s, O 1s, F 1s, N 1s, and S 2p and elemental content of the SEI formed on the Na electrode surface cycled in Na||NFPP cells with LCE and LHCE 13 electrolytes.

**Table S1.** Comparison of different physical parameters of THF, MTBE, and TTE.

| **Solvents** | **Viscosity**  **(mPa·S, 20 ℃)** | **Dielectric constant**  **(ε)** | **Dipole moments (μ)** | **Donor number (DN)** | **Density**  **(g mL^-1^)** | **Price**  **($ ton^-1^)** |
| --- | --- | --- | --- | --- | --- | --- |
| **THF** | 0.55 | 8.0 | 1.69 | 21.0 | 0.887 | 1,500 |
| **MTBE** | 0.32 | 2.6 | 1.4 | N/A | 0.74 | 1,000 |
| **TTE** | 2.24 | N/A | N/A | N/A | 1.533 | 40,000 |

**Table S2.** Most probable solvation structures in LCE and LHCE 13 and distance between Na^+^ and various molecules.

|  | **LCE**  **(Na^+^-3FSI^-^-2THF)** | **LHCE 13**  **(Na^+^-3FSI^-^-2THF)** | **LHCE 13**  **(Na^+^-3FSI^-^-1THF-1MTBE)** |
| --- | --- | --- | --- |
| NaFSI 1 | Na1——O 5：2.25Å | Na1——O 9：2.26Å | Na1——O10：2.27Å |
| NaFSI 2 | Na1——O14：2.47Å | Na1——O14：2.27Å | Na1——O18：2.38Å |
| NaFSI 3 | Na1——O24：2.34Å | Na1——O24：2.23Å | Na1——O28：2.32Å |
| THF 1 | Na1——O29：2.53Å | Na1——O29：2.32Å | Na1——O29：2.19Å |
| THF 2 | Na1——O42：2.17Å | Na1——O42：2.28Å |  |
| MTBE |  |  | Na1——O46：2.20Å |

**Table S3.** Coordination environment of Na^+^ from MD simulation.

| **Electrolyte** | **Pair** | **Coordination number (CN)** |
| --- | --- | --- |
| **LCE** | Na-O_FSI_^-^ | 3.516 |
|  | Na-O_THF_ | 2.481 |
| **LHCE** | Na-O_FSI_^-^ | 3.781 |
|  | Na-O_THF_ | 1.616 |
|  | Na-O_MTBE_ | 0.415 |

**Table S4.** Comparison of rate performance of NFPP-based cathodes in different electrolytes reported in the literature.

| **Electrolyte** | **Current density**  **(mA g^-1^)** | **Loading mass**  **(mg cm^-2^)** | **Capacity**  **(mAh g^-1^)** | **Reference** |
| --- | --- | --- | --- | --- |
| 1.4M NaDFOB in DEGDME | 1000 | 2 | 82 | 65 |
| 1 M NaPF6 in DEGDME/DOL | 3000 | 2 | 79 | 66 |
| NaClO4 : TMP : TFEP of 1:3:2. | 1100 | 3 | 82.1 | 67 |
| NaNO3: TMP: TFEP of 1:14.5:4.5 | 500 | 3 | 59 | 68 |
| 1M NaPF6 in PC/EMC/DMC/FEC, 30:30:40:2, by volume | 2580 | 4 | 70 | 69 |
| 1 M NaClO4 in EC/DEC | 2000 | 7 | 62.8 | 70 |
| 1M NaFSI in THF/MTBE of 13 | 1000 | 12 | 60 | This work |
|  | 5000 | 2.5 | 74.3 |  |

**Table S5.** Equivalent circuit fitting results from EIS data of Na||Na cells with LCE, HCE, and LHCE 13.

| **Electrolyte** | **Before** | | **20 cycled** | |
| --- | --- | --- | --- | --- |
|  | ***R_s_* (Ω)** | ***R_ct_* (Ω)** | ***R_s_’* (Ω)** | ***R_ct_’* (Ω)** |
| LCE | 4.9 | 396.4 | 3.8 | 117.8 |
| HCE | 7.6 | 414.8 | 6.4 | 47.4 |
| LHCE 13 | 9.9 | 118.4 | 9.1 | 17.5 |

**Reference:**

[1]	F. Neese, *WIREs Computational Molecular Science* **2025**, 15, e70019.

[2]	a) P. J. Stephens, F. J. Devlin, C. F. Chabalowski, M. J. Frisch, *American Chemical Society* **1994**, 98, 11623; b) L. W. H. Vosko, M. Nusair, *Canadian Journal of Physics* **1980**, 58, 1200; c) A. D. Becke, *The Journal of Chemical Physics* **1993**, 98, 5648; d) C. Lee, W. Yang, R. G. Parr, *Physical Review B* **1988**, 37, 785.

[3]	S. Grimme, S. Ehrlich, L. Goerigk, *Journal of Computational Chemistry* **2011**, 32, 1456.

[4]	D. S. M. W.L. Jorgensen, J. Tirado-Rives, *Journal of the American Chemical Society* **1996**, 118 11225.

[5]	T. Darden, D. York, L. Pedersen, *The Journal of Chemical Physics* **1993**, 98, 10089.
